# Evolution of Public Attitudes and Opinions Regarding COVID-19 Vaccination During the Vaccine Campaign in China: Year-Long Infodemiology Study of Weibo Posts

**DOI:** 10.2196/42671

**Published:** 2023-02-16

**Authors:** Yimin Hong, Fang Xie, Xinyu An, Xue Lan, Chunhe Liu, Lei Yan, Han Zhang

**Affiliations:** 1 School of Health Management China Medical University Shenyang China; 2 Medical Basic Experimental Teaching Center China Medical University Shenyang China

**Keywords:** COVID-19 vaccines, social media, infodemiology, sentiment analysis, opinion analysis, monitoring public attitude, gender differences, LDA, COVID-19

## Abstract

**Background:**

Monitoring people’s perspectives on the COVID-19 vaccine is crucial for understanding public vaccination hesitancy and developing effective, targeted vaccine promotion strategies. Although this is widely recognized, studies on the evolution of public opinion over the course of an actual vaccination campaign are rare.

**Objective:**

We aimed to track the evolution of public opinion and sentiment toward COVID-19 vaccines in online discussions over an entire vaccination campaign. Moreover, we aimed to reveal the pattern of gender differences in attitudes and perceptions toward vaccination.

**Methods:**

We collected COVID-19 vaccine–related posts by the general public that appeared on Sina Weibo from January 1, 2021, to December 31, 2021; this period covered the entire vaccination process in China. We identified popular discussion topics using latent Dirichlet allocation. We further examined changes in public sentiment and topics during the 3 stages of the vaccination timeline. Gender differences in perceptions toward vaccination were also investigated.

**Results:**

Of 495,229 crawled posts, 96,145 original posts from individual accounts were included. Most posts presented positive sentiments (positive: 65,981/96,145, 68.63%; negative: 23,184/96,145, 24.11%; neutral: 6980/96,145, 7.26%). The average sentiment scores were 0.75 (SD 0.35) for men and 0.67 (SD 0.37) for women. The overall trends in sentiment scores showed a mixed response to the number of new cases and significant events related to vaccine development and important holidays. The sentiment scores showed a weak correlation with new case numbers (R=0.296; *P*=.03). Significant sentiment score differences were observed between men and women (*P*<.001). Common and distinguishing characteristics were found among frequently discussed topics during the different stages, with significant differences in topic distribution between men and women (January 1, 2021, to March 31, 2021: χ^2^_3_=3030.9; April 1, 2021, to September 30, 2021: χ^2^_4_=8893.8; October 1, 2021, to December 31, 2021: χ^2^_5_=3019.5; *P*<.001). Women were more concerned with side effects and vaccine effectiveness. In contrast, men reported broader concerns around the global pandemic, the progress of vaccine development, and economics affected by the pandemic.

**Conclusions:**

Understanding public concerns regarding vaccination is essential for reaching vaccine-induced herd immunity. This study tracked the year-long evolution of attitudes and opinions on COVID-19 vaccines according to the different stages of vaccination in China. These findings provide timely information that will enable the government to understand the reasons for low vaccine uptake and promote COVID-19 vaccination nationwide.

## Introduction

The COVID-19 pandemic, with its global spread and unique transmission mode, has posed an unprecedented threat to the lives and health of people across the globe. By March 2022, COVID-19 had infected more than 479 million people, and more than 6 million deaths had been reported worldwide [1]. However, no specific cure exists for this disease, making vaccines the most effective way to reduce its spread [2]. Several countries, including the United States, the United Kingdom, and China, have launched mass vaccination programs to attain herd immunity. However, prior research has shown that some people hesitate or refuse to be vaccinated against COVID-19 [3]. According to a World Health Organization (WHO) announcement in 2019 [4], vaccine hesitancy is one of the top threats to global health. Public attitudes often determine compliance with protective measures such as vaccination. Numerous studies have investigated the impact of attitudes on subsequent behaviors (such as undertaking COVID-19 testing [5] and mask wearing [6,7]). These studies have suggested that understanding people’s perspectives could help health authorities design robust interventions and policies to respond to COVID-19.

The opinions shared on social media platforms strongly influence people’s decisions. Information passed through the media impacts users’ willingness to be vaccinated [8]. Facilitated by social media, public emotions and concerns can be evaluated using online discussions or posts [9-11]. During the COVID-19 pandemic, numerous researchers collected social media data to understand public opinion on COVID-19 vaccines. However, most published studies include data collected during a limited period [12-14] or else before or at the beginning of vaccination [15-17]. There have been few studies of postvaccination opinions in the general population. Monitoring the public’s attitude toward vaccines before or at the beginning of vaccination can provide valuable information to predict people’s acceptance of vaccines. However, decision-making on immunization involves weighing the risks and benefits of vaccinating and considering others’ advice and experiences [18]. People tend to adapt their behavior, revise their judgments, and make decisions based on the information they receive, especially feedback from others’ experiences [19]. Therefore, it is valuable for health care providers to recognize how the public responds after vaccination and design vaccine-promoting strategies accordingly.

Moreover, people’s attitudes and opinions on vaccination can change over time [20,21]. Thus, a long-term study is required, not only to provide a broad picture of public discussion and attitudes regarding COVID-19 vaccines but also to detect the changes in sentiments and major topics of discussion during vaccination of the general population [22].

The Chinese government adopted a step-by-step vaccination strategy targeting “key groups” at high risk of exposure to the virus first, followed by all adults and, finally, children. The first dose of the COVID-19 vaccine was administered on December 31, 2020. By the end of 2021, over 2.8 billion vaccine doses had been administered in China, allowing the country to achieve a relatively high vaccination coverage rate [23]. However, an early study showed high hesitancy toward COVID-19 vaccination across metropolises in 5 countries, including China [13].

Gender is crucial in understanding the response to vaccination. The importance of including gender-differentiating factors in investigating barriers to vaccination has been stressed by some authors [24]; there is evidence of a gender gap in hesitance to be vaccinated not only against COVID-19 [25,26] but also against other disease [27]. Women have a central role in ensuring the health of their children [28] and are at high risk of contracting the disease, as they are more likely to work in the health care and education sectors [26]. In contrast, men have been reported to be less likely than women to comply with recommended or mandated behavioral measures (eg, home quarantine and mask wearing) that put them in greater danger [29]. The Madrid Statement, released by the WHO in 2001, clearly states that, in order to achieve the highest standard of health, policy makers must recognize that men and women have different needs, obstacles, and opportunities. Therefore, it is crucial to examine whether gender differences influence concerns about vaccines and design gender-based public health policies accordingly.

The objectives of this study were to (1) monitor the public attitude regarding COVID-19 vaccination and how it changed over time during the vaccination campaign in China; (2) reveal how public opinion on COVID-19 vaccines evolved over the course of the campaign by extracting topics from online posts; and (3) discover whether users of different genders were more likely to post on particular topics and, thus, identify gender-specific patterns in attitudes and opinions regarding COVID-19 vaccines.

## Methods

### Data Collection and Preprocessing

**Figure 1:**
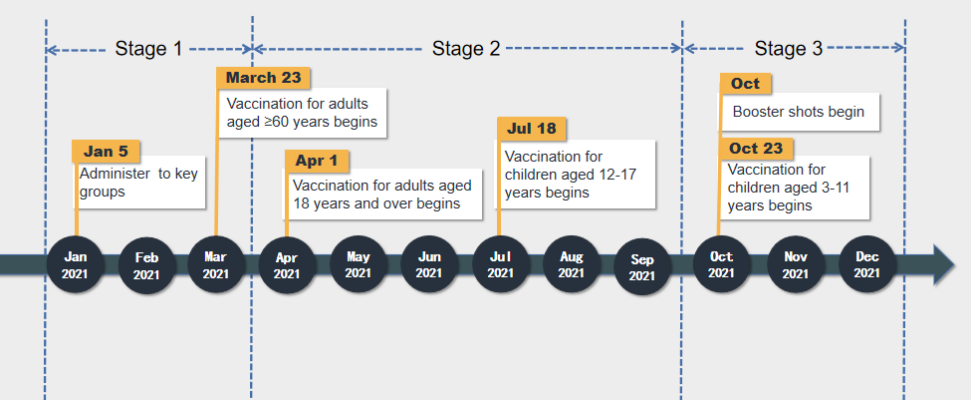
Timeline of the COVID-19 vaccination campaign in China.

Posts related to COVID-19 vaccination were collected from Sina Weibo, the largest and one of the most influential social media platforms in China: It had 584 million monthly active users in 2022 [30]. A web crawler tool was used to search for predefined keywords, including “COVID-19 vaccine”(新冠疫苗), “SINOVAC vaccine” (科兴疫苗), “Sinopharm vaccine” (国药疫苗), “Pfizer vaccine” (辉瑞疫苗), and “Moderna vaccine” (莫德纳疫苗). The SINOVAC and Sinopharm vaccines are China’s homemade COVID-19 vaccines and account for the majority of shots given in China. Because of the limitations of the Weibo search application programming interface (API), our web crawler obtained only the latest 50 pages of the search content. The time frame for the crawled data was from January 1, 2021, to December 31, 2021; this covered the entire vaccination process, from the first shots until a few months after booster shots began to be administered (a detailed timeline of the progress of COVID-19 vaccination in China is presented in Figure 1). Information collected about each crawled post included the user ID; post text; posting date; user gender; geolocation; and number of retweets, comments, and favorites.

In contrast to individual accounts, Weibo’s official accounts (eg, media or government accounts) focused on producing and disseminating vaccination knowledge and news. Therefore, to obtain the general public’s genuine attitude toward vaccines, only original posts by individual users were included for further analysis; posts from official accounts and news stories retweeted by individuals were excluded.

Before analysis, the crawled data were preprocessed to remove URLs, duplicate posts, and punctuation. Subsequently, we used Jieba [31], a word segmentation and part-of-speech tagging tool for Chinese text analysis, to process the data. Further, we applied a stop word list to eliminate meaningless stop words.

### Data Analysis

#### Sentiment Analysis

Text sentiment analysis is a text classification method that detects the positive or negative sentiment polarity of a given text. The techniques used for sentiment classification can be roughly divided into machine learning, lexicon-based, and hybrid approaches [32]. In this study, the SnowNLP tool [33], a Python kit, was used to analyze the sentiment polarity of each Weibo post, aided by lexicons. Subsequently, the naive Bayes algorithm, a machine learning approach, was used for text classification. SnowNLP was developed for sentiment analysis of Chinese texts and generates a sentiment score ranging from 0 to 1. It has been used to measure public opinion in areas including e-commerce [34] and public health events [35]. A post was considered positive if the sentiment score was greater than 0.6 and negative if it was smaller than 0.4; otherwise, the score was considered neutral [35-37]. Finally, the percentage of posts in each of the 3 sentiment categories was calculated. To detect temporal trends, we monitored the weekly changes in the proportions of positive, negative, and neutral posts. The weekly variations in the average sentiment score for men and women and the longitudinal changes between the sentiment score and the number of new cases were also registered.

#### Thematic Analysis

Thematic analysis is an effective method for discovering hidden topics in textual discourse. We used latent Dirichlet allocation (LDA), a popular topic modeling algorithm proposed by Blei et al in 2003 [38], to extract public opinions from posts. LDA is an unsupervised text classification algorithm based on a 3-layer Bayesian structure that maps the relationship between topics, documents (posts in this study), and words [39].

First, we sorted all the posts chronologically to show how public opinion on COVID-19 vaccination evolved. Subsequently, we classified them into 3 stages, based on China’s inoculation timeline (Figure 1).

Stage 1 lasted from January 1, 2021, to March 31, 2021; this stage signaled the beginning of the COVID-19 vaccination of some key population groups with a high infection risk [40]. These groups included workers in the cold-chain industry, customs, health care, markets, and public transport, as well as personnel traveling abroad. In total, 119.82 million doses were administered during this period [41].

Stage 2 lasted from April 1, 2021, to September 30, 2021; this stage covered the first and second vaccine doses for all eligible adults, as well as children 12 years to 17 years of age [42]. A total of 2.09 billion doses were administered in this stage.

Stage 3 lasted from October 1, 2021, to December 31, 2021; the general public received the booster dose at this stage. This period also covered the beginning of vaccination of children who were 3 years to 11 years of age [43]. In total, 623.88 million doses were administered in stage 3.

Then, we performed an LDA analysis of the posts published during each stage. A key concern in running LDA was determining the number of topics. Thus, topic models with different numbers of topics were compared using perplexity and coherence metrics: Lower perplexity and higher coherence indicate a better LDA model. By combining expert opinions with the values of perplexity and coherence, we chose the optimal number of topics for the LDA model at each stage. We further explored the differences in opinions on COVID-19 vaccination for men and women at each stage.

## Results

### Descriptive Results

**Figure 2:**
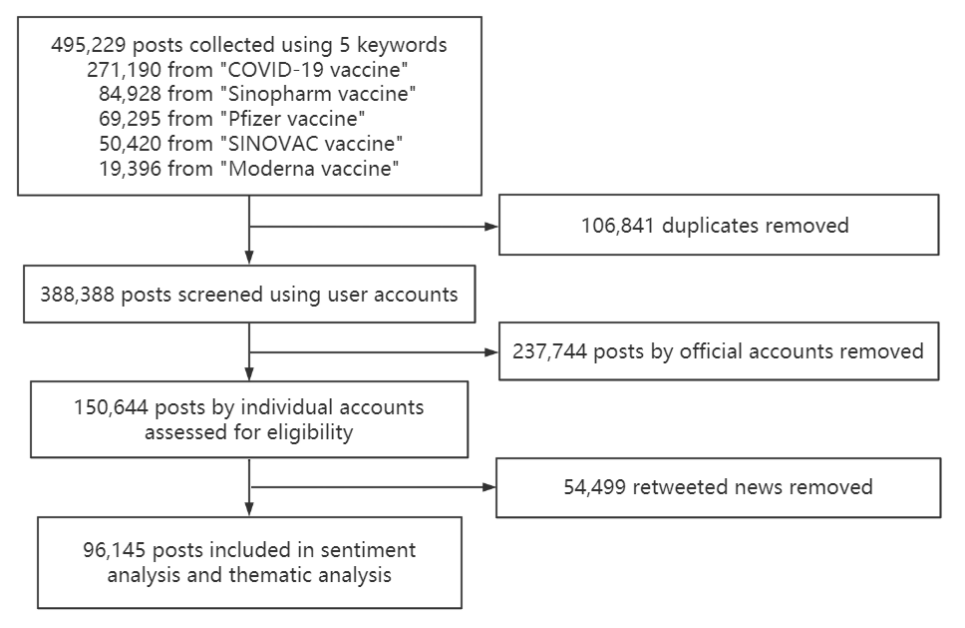
Flow diagram for the selection of posts.

The search identified 495,229 posts. After excluding 106,841 duplicates, 388,388 posts remained. We further eliminated 237,744 posts from official accounts and 54,499 news posts retweeted by individual users. A flow diagram that describes the inclusion process is shown in Figure 2. In total, 96,145 original posts by 59,699 individual users were retained for further analysis (refer to Multimedia Appendix 1 for the distribution of Weibo users’ followers). These posts attracted 235,645 comments, 373,093 favorites, and 36,074 retweets, which indicate the potential influence of the posts on Weibo.

**Figure 3:**
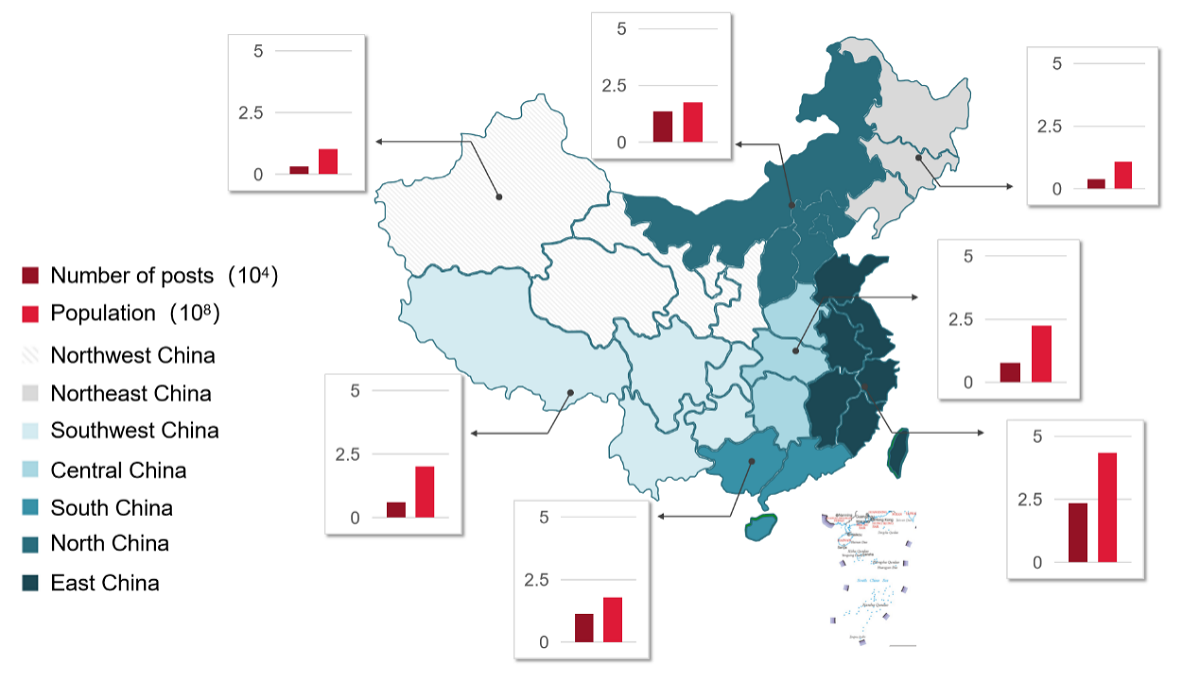
Distribution of posts by individual users, with the population sizes of 7 geographic regions of China.

The gender composition of the posters was 37.37% (35,934/96,145) male and 62.63% (60,211/96,145) female. There were 18,462 posts that secured the user’s location and 8215 posts from overseas. The remaining 69,468 posts covered all 34 divisions of China. The geographical distribution of posts and the size of the population in each region are presented in Figure 3. We grouped the 34 divisions into 7 regions based on provincial borders and administrative areas for clarity and readability. A complete list of the divisions included in each region is available in Multimedia Appendix 2. Highly populated regions such as the east yielded denser distributions, and less populated places such as the southwest region yielded less dense distributions. Pearson correlation was conducted to explore the relationship between the population size and number of posts at the divisional level, and a significantly positive correlation was observed (*r*=0.586, *P*<.001).

### Sentiment Polarity of Vaccine-Related Posts

In general, positive sentiment regarding the COVID-19 vaccine was dominant on Weibo, with 65,981 (65,981/96,145, 68.63%) positive posts, 23,184 (23,184/96,145, 24.11%) negative posts, and 6980 (6980/96,145, 7.26%) neutral posts. The average sentiment scores were 0.75 (SD 0.35) and 0.67 (SD 0.37) for men and women, respectively, and the weekly sentiment of women was consistently lower than that of men except in 1 week (refer to Multimedia Appendix 3). We used an independent samples *t* test to compare the daily averaged sentiment scores for men and women and found significant differences in sentiment between the 2 groups (t_77,988.52_=34.34, *P*<.001). Posts with a positive attitude accounted for the largest proportion for both women and men (39,365/60,211, 65.38% vs 26,616/35,934, 74.07%), followed by negative attitudes (15,908/60,211, 26.42% vs 7276/35,934, 20.25%) and neutral attitudes (4938/60,211, 8.2% vs 2042/35,934, 5.68%).

### Evolution of Sentiment Scores

**Figure 4:**
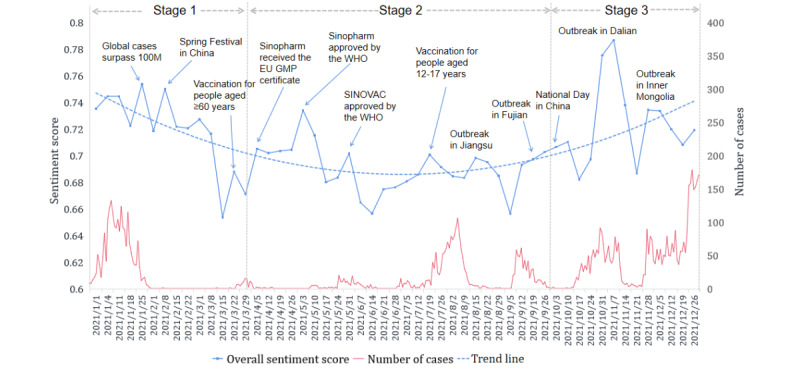
New cases and sentiment distribution by week. Significant events that correlated with the spikes in sentiment are also marked. EU: European Union; GMP: good manufacturing process; WHO: World Health Organization.

Figure 4 shows both the weekly averaged sentiment scores for all Weibo users and the number of newly confirmed COVID-19 cases. Although the overall trend of the sentiment scores fluctuated over time, the overall trend (blue dotted line in Figure 4) was at first downward but then went upward; the average score was 0.715 (SD 0.346) in stage 1, decreased to 0.690 (SD 0.369) in stage 2, and finally increased to 0.724 (SD 0.356) in stage 3.

The overall trend in sentiment also showed a certain degree of correlation with the number of new cases. The sentiment score was initially positive in January 2021, during which the outbreak occurred; it went downward from February 2021 to July 2021 when the number of new cases remained at a low level. As outbreaks occurred in several provinces from August 2021 to December 2021, positive sentiment appeared to increase. This fluctuating pattern suggests that people tended to express more positive sentiments toward vaccines when the pandemic became severe. Spearman correlations revealed a weak but significant positive correlation between the weekly cumulative number of new cases and the weekly average sentiment score (R=0.296; *P*=.03).

We further identified a number of events that may help explain the rises in positive sentiment from February 2021 to July 2021, when the number of new cases remained low. These events included important holidays and news about vaccines. For instance, users were highly excited and expressed strong positive sentiment around May 7, 2021, when the WHO approved China’s first domestic vaccine, the Sinopharm vaccine, for emergency use.

### Discussion Topics During Each Stage

**Figure 5:**
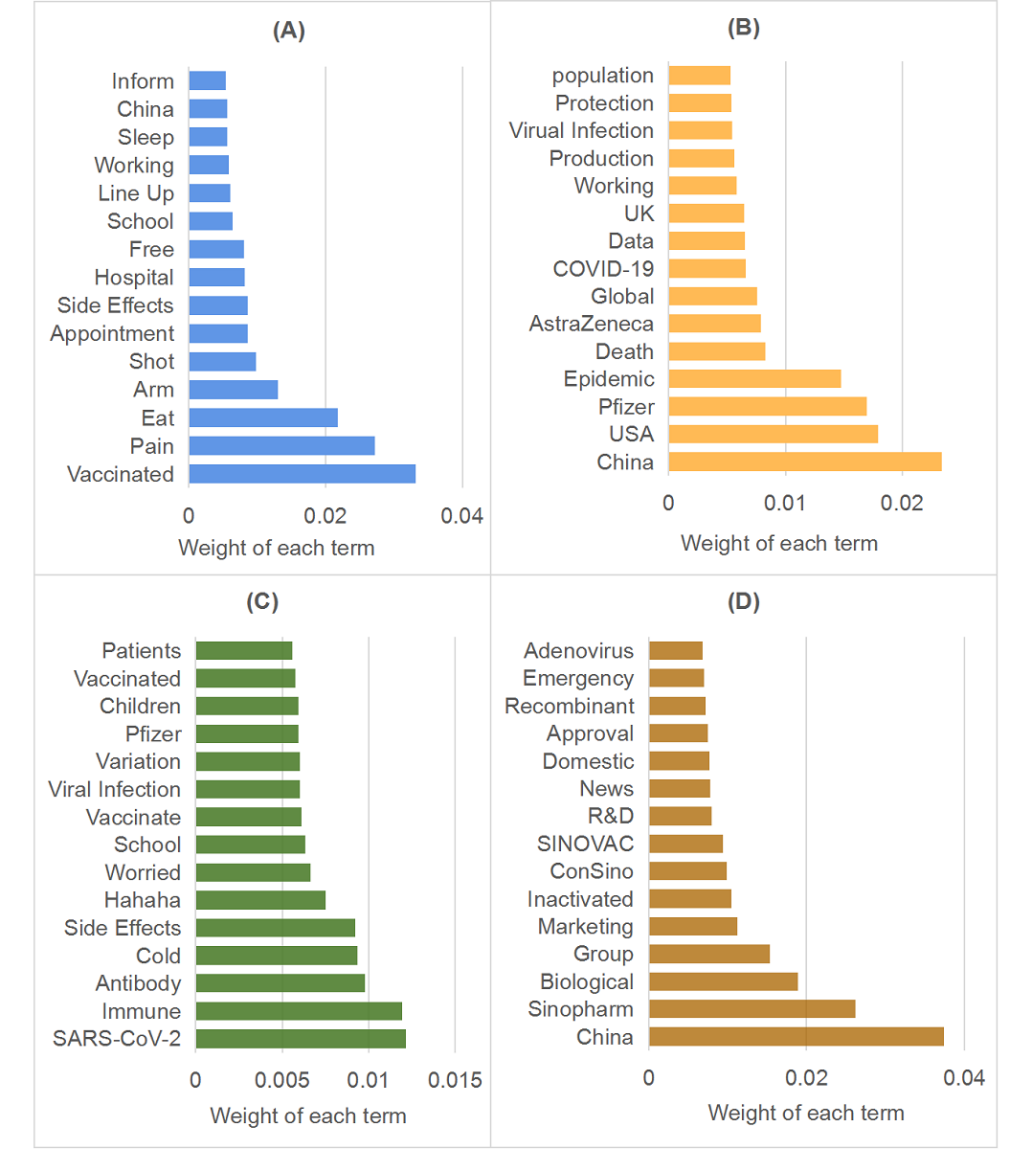
The 4 topics discussed during stage 1, with the most relevant terms: (A) topic 1: vaccine safety; (B) topic 2: foreign vaccines; (C) topic 3: vaccine effectiveness; (D) topic 4: domestic vaccine research and development (R&D).

**Figure 6:**
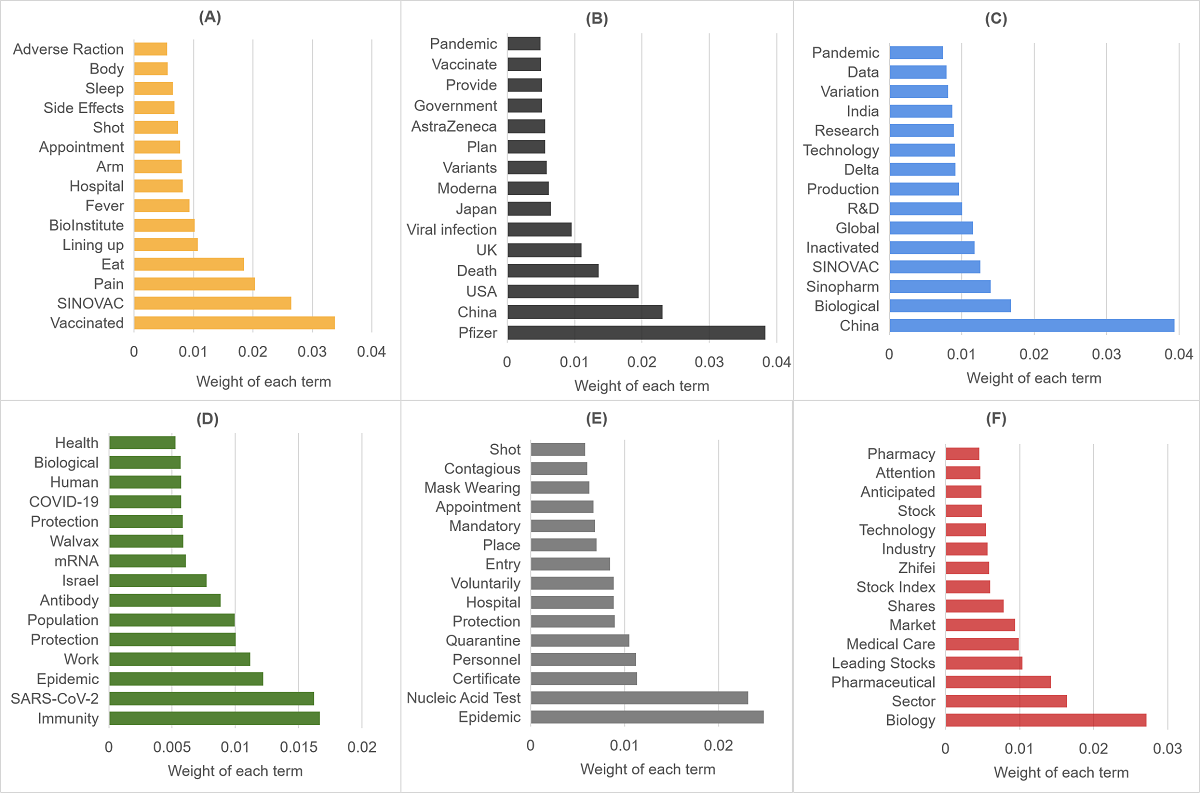
The 6 topics discussed during stage 2, with the most relevant terms: (A) topic 1: vaccine safety; (B) topic 2: global pandemic situation; (C) topic 3: Delta variant; (D) topic 4: vaccine effectiveness; (E) topic 5: pandemic control; (F) topic 6: stock market. R&D: research and development.

**Figure 7:**
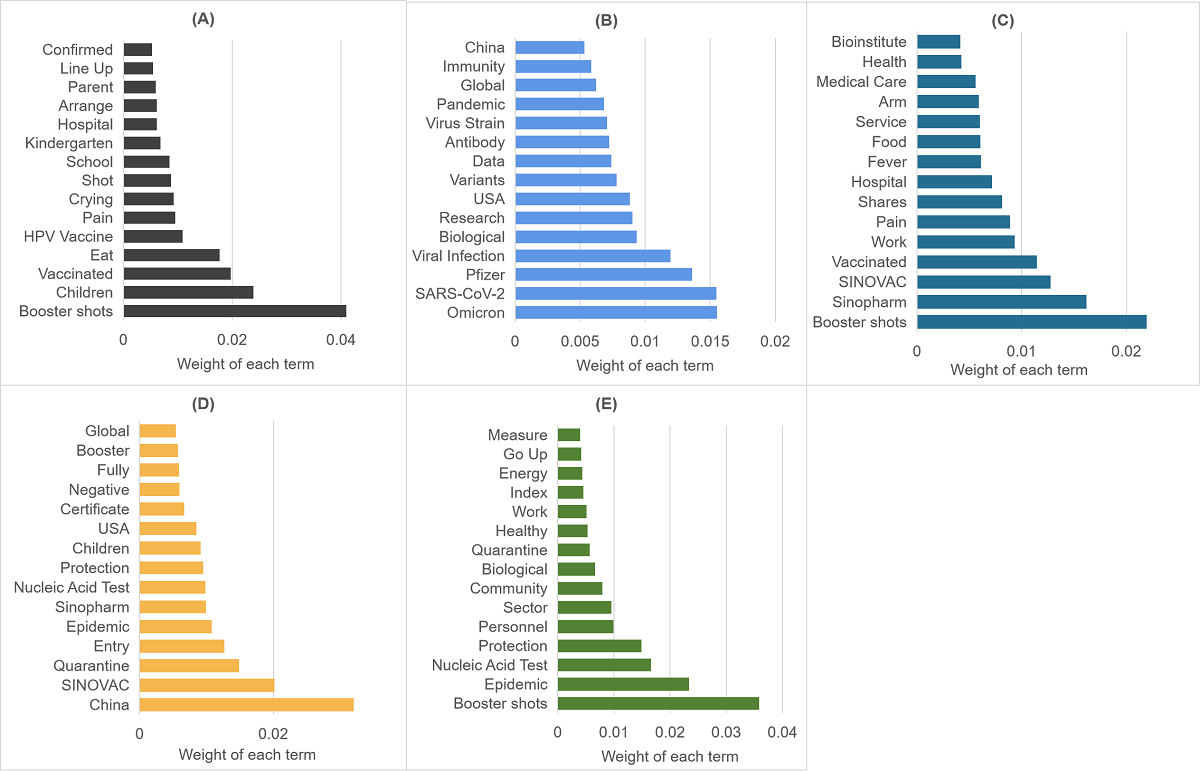
The 5 topics discussed in stage 3, with the most relevant terms: (A) topic 1: vaccine safety for family; (B) topic 2: Omicron variant; (C) topic 3: safety of the booster shot; (D) topic 4: entry policy; (E) topic 5: prevention and control. HPV: human papillomavirus.

The LDA analysis allowed us to identify 4 topics from 17,352 (17,352/96,145, 18.05%) posts in stage 1; 6 topics from 57,632 (57,632/96,145, 59.94%) posts in stage 2; and 5 topics from 21,161 (21,161/96,145, 22.01%) posts in stage 3. Topics across all stages are summarized in Figures 5-7, with the 15 most relevant terms for each.

In stage 1, the most dominant topic was the safety of COVID-19 vaccines (8392/17,352, 48.36%). Although excited about the conditional approval of domestic vaccines for general use, people were concerned about vaccine safety. Adverse effects, such as arm pain, headache, sleepiness, and fatigue, were reported frequently. The second most dominant topic involved vaccines produced by foreign companies (4293/17,352, 24.74%). As some foreign vaccines (eg, those from Pfizer, Moderna, and AstraZeneca) were granted approval for use in many countries, the public paid close attention to their efficacy rates, their safety for children and older adults, and deaths or positive tests after vaccination with them. News about massive COVID-19 vaccine rollouts in other countries (eg, the United Kingdom and United States) also gained the public’s attention. In addition, the effectiveness of vaccines (2540/17,352, 14.64%) was another hot topic: There were discussions of whether the vaccines could develop sufficient antibodies against the virus, the durability of vaccine-mediated immunity against new variants, and the possibility of future infection as a consequence of waning immunity. The last topic involved the progress of vaccine research and development (R&D) in China (2127/17,352, 12.26%). The public was excited by the news that COVID-19 vaccines developed by Chinese firms Sinopharm and SINOVAC had been approved for general use by the Chinese National Medical Products Administration. The data showed the efficacies of these 2 inactivated vaccines were compatible with WHO requirements [44].

In stage 2, safety of the COVID-19 vaccines was still the most dominant topic (32,662/57,632, 56.67%). By the end of this period, China had administered 2.09 billion doses of its homegrown vaccines, the majority of which were developed by state-owned companies SINOVAC and Sinopharm. Thus, the users were now discussing what they had experienced after vaccination. Meanwhile, the global pandemic situation of COVID-19 (8557/57,632, 14.85%) was of great concern to the public. People talked about the deaths caused by the pandemic and the progress of COVID-19 vaccination in other countries (eg, the United States, the United Kingdom, and India). In addition, because of the rising number of COVID-19 cases in Japan and that country’s slow-moving vaccination program, concerns were raised regarding the safety of conducting the Olympic Games in Tokyo and the possibility that Olympic-related infections might spread throughout Japan as well as globally. The Delta variant (5587/57,632, 9.69%) was another topic that attracted Weibo users’ attention. The WHO indicated in June 2021 that Delta was becoming the dominant COVID-19 variant worldwide. Thus, some users discussed whether the existing vaccines could effectively protect them from reinfection. Nevertheless, they also expressed confidence in the vaccines when most cases of reinfection proved to be mild or asymptomatic. Furthermore, there were discussions comparing the safety and effectiveness of inactivated and mRNA vaccines. In general, vaccination effectiveness (4246/57,632, 7.37%) remained a popular topic. Users wondered whether and when detectable antibodies could be produced in the population after the initial dose and if the current vaccines would continue to be protective against new COVID-19 variants. Pandemic control measures also generated considerable interest (4012/57,632, 6.96%). As the Delta variant of the coronavirus spread worldwide, China experienced several pandemic outbreaks caused by it. To battle the pandemic, the nucleic acid test and proof of vaccination were required in various circumstances, such as entering the country; Weibo users showed tolerance and understanding of such prevention and control measures and quarantine policies. How the stock markets responded to the vaccination progress and pandemic situation was the last topic (2568/57,632, 4.46%). The users concentrated on stock market volatility and perceived a positive impact from COVID-19 vaccination on the profitability of the biopharmaceutical sector. The news that the Biden administration supported waiving intellectual property protection for COVID-19 vaccines also gained users’ attention.

In stage 3, the most dominant topic was vaccine safety for family members (7934/21,161, 37.49%). In late October, vaccines were administered to children aged 3 years to 11 years and people with underlying conditions. The public asked for information regarding the safety and side effects of vaccination in children and older parents. Users also expressed concerns about adverse effects and reduced effectiveness due to receiving human papillomavirus and COVID-19 vaccines simultaneously or after a short interval. The second most dominant topic was the Omicron variant (4244/21,161, 20.06%), which had replaced the Delta variant in December as being responsible for most COVID-19 cases globally. Although no evidence nor data suggested the failure of existing vaccines, the public was still highly concerned about their effectiveness against the Omicron variant. As a considerable number of Chinese people were fully vaccinated during stage 3, the safety of booster shots (3897/21,161, 18.42%) was also widely discussed, along with the safety of mixing and matching COVID-19 vaccine booster doses and eligibility for the booster dose. The next topic centered on the nation’s entry policy (3009/21,161, 14.22%). To boost economies suffering from COVID-19 lockdowns, various countries, such as Australia [45], the United States [46], and the United Kingdom, reopened their borders in November 2021. In particular, many countries allowed international visitors to enter quarantine-free conditions in November 2021 under certain conditions, including being fully vaccinated or offering proof of a negative nucleic acid test. The users, especially those who wanted to study abroad, were excited about the news that SINOVAC and Sinopharm were recognized by many countries as approved vaccinations for quarantine-free travel. The last topic was related to the role of the prevention and control policy in curbing the COVID-19 pandemic (2077/21,161, 9.82%). With COVID-19 vaccines being distributed worldwide, reports that vaccination was effective in reducing new case numbers and deaths attracted the public’s attention; the users also discussed whether the policy of prevention and control could be changed under a condition of high vaccination coverage.

### Gender Differences Across Topics

**Figure 8:**
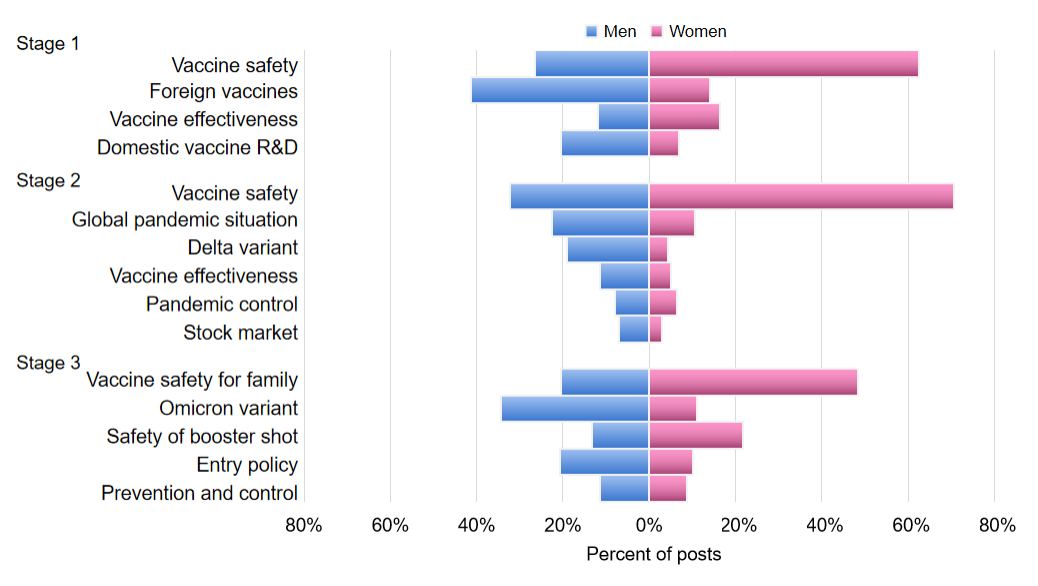
Difference in opinion and attention regarding COVID-19 vaccines between men and women over time. The topics are sorted in descending order of the proportion of posts in each stage. R&D: research and development.

Figure 8 shows the proportions of posts on each topic written, divided by men and women. In all 3 stages, there was a clear gender gap in concerns about the COVID-19 vaccination. We examined the difference between the topic distribution for men and women using the chi-square test (refer to Multimedia Appendix 4 for the number of posts and sentiment scores for both men and women). The results showed that the 2 groups differed significantly at each stage (stage 1: χ^2^_3_=3030.9; stage 2: χ^2^_4_=8893.8; stage 3: χ^2^_5_=3019.5; *P*<.001). Vaccine safety was the most dominant concern of women during all 3 stages, followed by vaccine effectiveness in stage 1. In contrast, men more frequently discussed foreign brands of vaccine and domestic vaccine R&D in stage 1; the global pandemic situation, Delta variant, vaccine effectiveness, pandemic control, and the stock market in stage 2; and the Omicron variant, entry policy, and prevention and control policy in stage 3.

## Discussion

### Principal Findings

This study affords insight into how public attitudes and opinions evolved over the course of the vaccination campaign in China and provides a unique source of data for understanding gender differences in COVID-19 vaccine hesitancy. We found that positive attitudes were dominant in posts and that public sentiment could be affected directly by new cases and major events. By using topic modeling, we found a shift in public interest, with users focusing on domestic vaccine R&D initially and then paying more attention to epidemiological (the pandemic situation, pandemic control, and Delta and Omicron variants) and economic (the stock market and entry policy) issues. However, the safety and effectiveness of the vaccine were perennial concerns of users throughout the whole vaccination campaign. In addition, we also found significant differences in both sentiment and topics between men and women. Posts by women focused on vaccine safety and side effects of vaccination, whereas those by men addressed a broader range of topics including vaccine development, the global pandemic situation, COVID’s impact on the economy, and domestic pandemic control policy.

### Trend in Sentiment Scores With Confirmed Case Numbers and Significant Events

Using sentiment analysis, we found positive attitudes were typical in posts, suggesting that Weibo users had confidence in domestically made COVID-19 vaccines. Nevertheless, overall public sentiment fluctuated over time. The observed trend showed a mixed response to changes in the number of cases and other significant events. Earlier studies reported a weak, but significant positive correlation between sentiment scores and confirmed COVID-19 case numbers [47]. Our results confirmed that the patterns of public sentiment changed in response to pandemic severity. For instance, several increases in sentiment score were concurrent with the outbreaks caused by the Delta variant in different provinces from mid-August 2021 to December 2021. Every time a COVID-19 epidemic occurred, people’s positive sentiment toward vaccination increased. A possible explanation is that people’s perceived fear of infection and sense of self-protection led to a positive attitude or attentiveness toward vaccines [8,48,49]. Even a regionally limited epidemic could increase positive expectations nationwide [50]. In addition, significant events, such as news about vaccine development and celebration of important holidays, were found to drive up positive sentiment. Although the confirmed cases remained at a low, steady level in April 2021 and May 2021, the public’s sentiment was affected positively by the news that Sinopharm’s vaccine received European Union good manufacturing policy certification and Sinopharm and SINOVAC were approved for emergency use by the WHO. That these 2 domestic vaccines were perceived as safe and effective by international authorities had a positive impact on the public sentiment toward vaccines. Furthermore, the expectation of family reunions during important holidays such as the Spring Festival also increased people’s intention to receive the COVID-19 vaccine. Other studies have also found that major spikes in positive sentiments correspond closely to major events related to vaccine development [22], important holidays [47], and governmental decisions [51].

### Evolution of Topics Across Different Stages

Staged thematic analysis of posts allowed us to assess how public opinion on vaccination has changed over the course of the vaccination campaign. First, we found that uncertainties about the safety and effectiveness of the COVID-19 vaccine were shared concerns across each stage. The most dominant topic in each stage was vaccine safety, including postvaccination side effects, the safety of vaccination under special circumstances (eg, while menstruating or while suffering from a cold or if subject to allergies), contraindications for children and older adults, and the safety of receiving heterologous vaccines in the first 2 doses and the booster dose.

According to our results, the symptoms that the public most complained about included headache, fatigue, arm pain, nausea, sleepiness, cough, and longer menstrual cycles. Symptoms including fever, running nose, and lack of appetite were reported less frequently, and more severe adverse effects were not reported at all. This is consistent with the findings that the side effects of the Sinopharm COVID-19 vaccine were predictable and nonserious in the United Arab Emirates [52,53], Thailand [54], and Bangladesh [55].

Effectiveness of the vaccine was another recurring concern. In the early stage of the campaign, it was extensively discussed; the reported 94% to 95% efficacy rate of vaccines made by Pfizer and Moderna [56] and cases of reinfection after vaccination attracted the public’s attention. However, after large-scale vaccination was launched in China and more people were vaccinated, the focus of the discussion turned to when the detectable antibody could be produced after vaccination, how long immunity would last, and the reliability of vaccines when faced with new variants.

Second, we found typical and distinguishing characteristics in the topics across stages. Although aligned with the vaccination process, the focus of public attention varied at each stage. In stage 1, when the vaccine supply was insufficient and vaccines were only administered in China to certain groups of people, the public’s attention focused on the progress of domestic vaccine R&D and the efficacy of vaccines produced by foreign companies. During this period, data reported from the vaccine trials, vaccine campaigns launched in other countries, and postvaccination deaths or reinfections involving any brand of vaccines could gain the users’ attention and sometimes influence their acceptance of COVID-19 vaccines. In stage 2, when vaccines were administered to the public on a large scale, the discussions focused on experiences after receiving the vaccines as well as the duration of the protective immunity. In addition, as the highly contagious Delta variant became predominant worldwide, it aroused deep concern about the global pandemic situation and attention to governmental pandemic control policies related to quarantine and isolation requirements, nucleic acid tests, and mask wearing. In stage 3, the more transmissible Omicron variant that significantly reduced the effectiveness of vaccines grabbed the public’s attention [57]. Much concern focused on whether the Omicron variant could escape antibody immunity induced by vaccination, whether the immunity induced by inactivated vaccines (with which most people in China had been vaccinated [58]) provides sufficient protection against Omicron, and whether booster shots could maintain immunity levels. Moreover, long-term lockdowns and restrictions on labor mobility and travel, including border closings, had produced significant economic effects in many countries [59,60]; therefore, people paid close attention to the economic damage caused by mobility reduction and to global economic recovery policy.

### Gender Differences in Perception of COVID-19 Vaccines

A significant difference in vaccine sentiment scores between the male and female groups was observed. The sentiment of women was consistently less positive than that of men on a weekly basis, which is in line with other studies finding that COVID-19 vaccine hesitancy was higher among women than among men [61,62]. Moreover, we found gender-specific patterns in vaccination opinions. The most prominent feature was that vaccine safety was the dominant concern of women across all stages of the Chinese vaccination campaign. Women frequently discussed the side effects they experienced after being vaccinated and the contraindications to vaccination. According to a survey on the side effects of the Sinopharm vaccine, women report more symptoms after vaccination than men do [52]. Another survey indicated that adverse events were more common in women [63]. This might be why women more often discussed the side effects of the vaccines in our study.

In addition, women were more concerned about vaccination safety for family members, such as unknown future effects of the vaccine on children and risks to older people with pre-existing diseases. The possible explanation of this could be their status as caregivers in the family: Women are typically responsible for the health of other family members. It has been reported that mothers were less willing than fathers to vaccinate their children against COVID-19, owing to the unknown safety of a new vaccine [64,65]. Understanding what effects their family might experience after vaccination (and why) would help ease these anxieties.

In contrast, men reported broader concerns involving the global pandemic situation (eg, new variants or border re-opening policy), vaccine development, and the economic effects of the pandemic. Most of the time in China, men are reportedly the breadwinner in a family [66,67]. Men who were currently working were inclined to be vaccinated because they feared losing their jobs if they became infected [68]. This fear may be reinforced by the economic insecurity caused by continuous pandemic outbreaks, the shutdown of the border [60], and the governmental pandemic control policy [69]; it may explain men’s positive sentiment toward vaccination and their attention to progress in vaccine R&D and the global pandemic situation.

### Implications and Recommendations

Developing population immunity with vaccines requires high compliance with vaccination recommendations; this is hindered by vaccine hesitancy or refusal [70]. Trust in information sources during a pandemic is fundamental for vaccine acceptance [71]. Individuals who received information from their health care provider or public health department were more likely to be vaccinated [27,72]. A survey in China reported that 92.1% of the participants would only receive the COVID-19 vaccine if were provided adequate information [15]. Our findings on evolving patterns of public opinion suggest that health authorities should be aware of the trend of public concerns in different phases of a vaccination campaign and, accordingly, implement targeted practical actions, including synchronizing the information flow, designing timely and effective communication campaigns, and releasing guidance and suggestions for concrete actions. Clear and consistent communication by government authorities is crucial for fostering public vaccine preference and confidence in vaccine programs [71]: Authorities should explain how vaccines work, their potential side effects and contraindications, what doses are needed for protection, and the relevance of population-wide coverage to achieving herd immunity.

Furthermore, effective campaigns should consider gender when designing vaccine uptake strategies. For example, our finding that vaccine safety for family members was the dominant concern of women suggests that this should be addressed early in vaccination campaigns for children and older adults. Information campaigns should use straightforward language to ensure that communications are understood and tailored to different audiences [71,73,74].

### Comparison With Prior Work

To the best of our knowledge, this study is the first to analyze public sentiments and perceptions on COVID-19 vaccines adhering to the staged process of the vaccination campaign using Chinese social media data. Most other studies using social media to examine public opinions of COVID-19 vaccines in different countries were conducted with data that were either collected before the national vaccination rollout [13,14,75] or did not cover the entire vaccination campaign [16,50,76]. Therefore, changes in public opinions at different points in the campaign could not be captured. Furthermore, to monitor the public’s genuine sentiments and opinions toward COVID-19 vaccines, we included only posts by individual Weibo users. Although a large number of posts were therefore excluded, our findings are more reflective of the general public’s real perception of COVID-19 vaccination than if all posts had been retained. Finally, gender differences have rarely been mentioned in staged theme analyses of COVID-19 vaccine, especially in China [77,78]. We performed an in-depth analysis of the differences in attitude toward COVID-19 vaccination between men and women. Thus, our findings can offer important insights for government and health authorities in designing staged, gender-disaggregated plans for vaccine promotion.

### Limitations

This study has several limitations. First, we classified sentiment polarity as positive, negative, or neutral; further efforts should be made to distinguish public sentiment in more specific dimensions (eg, joy, fear, anger, and anticipation [79]) to understand the different emotional tones expressed in the text. Second, Weibo was reported to have 584 million monthly active users in 2022 (over one-third of China’s total population) [30]. Nevertheless, our data set may be unrepresentative of certain groups, as Weibo users in China tend to be young and well-educated [80]. Third, owing to the anticrawling mechanism in Weibo, the amount of data included in this study was relatively small compared with that used in research on other social media platforms, such as Twitter [81]. In future work, data could be collected from multiple sources, such as WeChat, or include comments from the original posts. Finally, we did not collect the users’ COVID-19 vaccination status, as this was not in their profiles. Although China had achieved 89.63% vaccine coverage rates at the end of our data collection [82], we cannot make the assertion that every post included in this study was published by a vaccinated user.

### Conclusion

Vaccines are considered one of the most effective ways to contain the COVID-19 pandemic. However, vaccine hesitancy limits global efforts against the disease. By analyzing Weibo posts, we revealed the evolution of attitudes and opinions on COVID-19 vaccines during the vaccination process in China. The study also provided first-hand evidence of gender-specific patterns in attitudes toward vaccination. Our study showed that data from social media can help in understanding public attitudes toward vaccines and other measures to curb the spread of diseases. The findings could also help governments and public health authorities understand vaccine-related intentions and hesitancy in the general population, enabling more targeted and publicly acceptable strategies to alleviate public concerns and boost vaccination intention.

## Appendix

Multimedia Appendix 1 Distribution of Weibo users’ followers.

Multimedia Appendix 2 Distribution of the posts, with the population sizes of 7 geographic regions of China.

Multimedia Appendix 3 Trends in weekly sentiment for both women and men.

Multimedia Appendix 4 The number of posts and sentiment scores for both males and females.

## Abbreviations

API: application programming interface
LDA: latent Dirichlet allocation
R&D: research and development
WHO: World Health Organization
