# Supplementary material for: Evolution of Public Attitudes and Opinions Regarding COVID-19 Vaccination During the Vaccine Campaign in China: Year-Long Infodemiology Study of Weibo Posts
Source: J Med Internet Res. 2023 Feb 16;25:e42671. doi: 10.2196/42671 (PMC9937109; doi:10.2196/42671)
Supplement: Multimedia Appendix 1 [file jmir_v25i1e42671_app1.docx]

**Multimedia Appendix 1.**

**Distribution of Weibo users’ followers**

| **Interval**  **(No. of followers)** | **Frequency**  **(No. of users)** |
| --- | --- |
| 0-99 | 24,086 |
| 100-999 | 28,935 |
| 1,000-9,999 | 5,315 |
| 10,000-99,999 | 1,024 |
| 100,000-999,999 | 317 |
| 1,000,000- | 22 |
| Total | 59,699 |
