# Supplementary material for: Evolution of Public Attitudes and Opinions Regarding COVID-19 Vaccination During the Vaccine Campaign in China: Year-Long Infodemiology Study of Weibo Posts
Source: J Med Internet Res. 2023 Feb 16;25:e42671. doi: 10.2196/42671 (PMC9937109; doi:10.2196/42671)
Supplement: Multimedia Appendix 2 [file jmir_v25i1e42671_app2.docx]

**Multimedia Appendix 2.**

**Distribution of the posts , with the population sizes of seven geographic regions of China.**

| Region | Provinces | Population(×10⁴) | No. of posts |
| --- | --- | --- | --- |
| **Northeast China** | Heilongjiang | **10,836** | **3,897** |
|  | Jilin |  |  |
|  | Liaoning |  |  |
| **North China** | Beijing | **17,479** | **13,677** |
|  | Hebei |  |  |
|  | Inner Mongolia |  |  |
|  | Shanxi |  |  |
|  | Tianjin |  |  |
| **East China** | Anhui | **43,531** | **23,615** |
|  | Fujian |  |  |
|  | Jiangsu |  |  |
|  | Jiangxi |  |  |
|  | Shandong |  |  |
|  | Shanghai |  |  |
|  | Taiwan |  |  |
|  | Zhejiang |  |  |
| **South China** | Macao | **18,014** | **11,302** |
|  | Guangdong |  |  |
|  | Guangxi |  |  |
|  | Hainan |  |  |
|  | Hong Kong |  |  |
| **Central China** | Henan | **22,421** | **7,684** |
|  | Hubei |  |  |
|  | Hunan |  |  |
| **Northwest China** | Gansu | **10,279** | **3,160** |
|  | Ningxia |  |  |
|  | Qinghai |  |  |
|  | Shaanxi |  |  |
|  | Xinjiang |  |  |
| **Southwest China** | Guizhou | **20,208** | **6,133** |
|  | Sichuan |  |  |
|  | Tibet |  |  |
|  | Yunnan |  |  |
|  | Chongqing |  |  |
