# Supplementary figures and images for: Evolution of Public Attitudes and Opinions Regarding COVID-19 Vaccination During the Vaccine Campaign in China: Year-Long Infodemiology Study of Weibo Posts
Source: J Med Internet Res. 2023 Feb 16;25:e42671. doi: 10.2196/42671 (PMC9937109; doi:10.2196/42671)

**Multimedia Appendix 3.**

**Trends in weekly sentiment for both women and men**


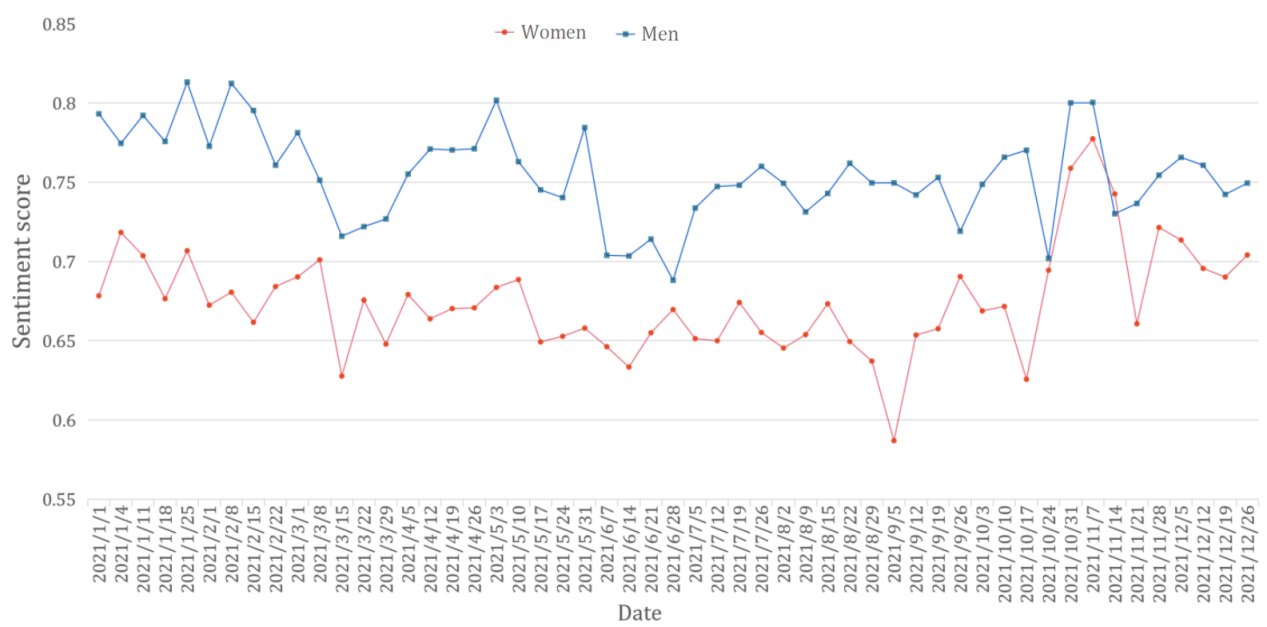

Supplement: Multimedia Appendix 3 [file jmir_v25i1e42671_app3.docx]
