# Supplementary material for: Evolution of Public Attitudes and Opinions Regarding COVID-19 Vaccination During the Vaccine Campaign in China: Year-Long Infodemiology Study of Weibo Posts
Source: J Med Internet Res. 2023 Feb 16;25:e42671. doi: 10.2196/42671 (PMC9937109; doi:10.2196/42671)
Supplement: Multimedia Appendix 4 [file jmir_v25i1e42671_app4.docx]

**Multimedia Appendix 4.**

**The number of posts and sentiment scores for both males and females**

| **Topic** | **Male** | | | **Female** | | | **χ2** | **P value** |
| --- | --- | --- | --- | --- | --- | --- | --- | --- |
|  | **Posts**  **(n)** | **Proportion**  **(%)** | **sentiment score** | **Posts**  **(n)** | **Proportion**  **(%)** | **sentiment score** |  |  |
| **Stage 1** | | | | | | | | |
| topic1 | 1812 | 26.50% | 0.76 | 6580 | 62.58% | 0.67 | 3030.85 | <.001 |
| topic2 | 2824 | 41.30% | 0.78 | 1469 | 13.97% | 0.70 |  |  |
| topic3 | 809 | 11.83% | 0.77 | 1731 | 16.46% | 0.67 |  |  |
| topic4 | 1392 | 20.36% | 0.77 | 735 | 6.99% | 0.70 |  |  |
| Stage 2 | | | | | | | | |
| topic1 | 4681 | 22.44% | 0.73 | 3876 | 10.54% | 0.65 | 8893.81 | <.001 |
| topic2 | 1456 | 6.98% | 0.77 | 1112 | 3.02% | 0.67 |  |  |
| topic3 | 6719 | 32.21% | 0.79 | 25943 | 70.55% | 0.67 |  |  |
| topic4 | 3964 | 19.01% | 0.76 | 1623 | 4.41% | 0.66 |  |  |
| topic5 | 2389 | 11.45% | 0.73 | 1857 | 5.05% | 0.66 |  |  |
| topic6 | 1648 | 7.90% | 0.77 | 2364 | 6.43% | 0.69 |  |  |
| Stage 3 | | | | | | | | |
| topic1 | 939 | 11.40% | 0.69 | 1138 | 8.81% | 0.71 | 3019.54 | <.001 |
| topic2 | 1691 | 20.52% | 0.87 | 6243 | 48.32% | 0.82 |  |  |
| topic3 | 1699 | 20.62% | 0.66 | 1310 | 10.14% | 0.64 |  |  |
| topic4 | 2824 | 34.27% | 0.72 | 1420 | 10.99% | 0.69 |  |  |
| topic5 | 1087 | 13.19% | 0.70 | 2810 | 21.75% | 0.71 |  |  |
